# Estimation of Alpha-Synuclein Monomer and Oligomer Levels in the Saliva of the Children With Autism Spectrum Disorder: A Possibility for an Early Diagnosis

**DOI:** 10.7759/cureus.9936

**Published:** 2020-08-22

**Authors:** Abubakkar Siddique, Humaira Fayyaz Khan, Shazia Ali, Athar Abdullah, Hina Munir, Madiha Ariff

**Affiliations:** 1 Physiology, Islamic International Medical College, Rawalpindi, PAK; 2 Physiology, Riphah University, Islamabad, PAK; 3 Internal Medicine, Dow University of Health Sciences, Karachi, PAK

**Keywords:** synuclein, alpha-synuclein monomer, alpha-synuclein oligomer, autism spectrum disorder, saliva

## Abstract

Background

In degenerative brain diseases like Parkinson's disease (PD), alpha-synuclein (a-syn) can be in its monomeric (a-syn-mono) or toxic oligomeric (a-syn-oligo) or as a total (a-syn-total) forms in the biological body fluids including saliva. Past research has observed major a-syn plasma variations in children with autism spectrum disorder (ASD) pointing toward brain degenerative components in their pathophysiology. No prior study has shown a-syn levels in ASD patients' saliva.

Objective

This study estimates the levels of alpha-synuclein monomer (a-syn-mono) and alpha-synuclein oligomer (a-syn-oligo) in the saliva of ASD affected children so that saliva can be a method for detecting disorder.

Materials and methods

This cross-sectional, multi-center study was conducted in Islamic International Medical College, Autism Resource Centre (ARC), and Step-to-learn Rehabilitation center for the slow learner in Rawalpindi. The research was performed for one year from August 2018 to August 2019. Saliva samples from 80 children (40 ASD affected children, and 40 age- and sex-comparable healthy controls) were collected. Specific anti-alpha-synuclein monomers (anti-a-syn-mono) and anti-alpha-synuclein oligomers (anti-a-syn-oligo) enzyme-linked immunosorbent assay (ELISA) kits analyzed the salivary samples. Mean ± SD were reported for quantitative data. The data between the two groups were compared using an independent t-test. The p-value of ≤ 0.05 was considered statistically significant.

Results

A total of 80 children were included in the study (n=40 ASD affected, n=40 healthy controls). The age of participating children was between four and eight years. The mean alpha-synuclein monomer level in the saliva of ASD children was 92.03 ± 117.09 pg/ml (p≤0.05), and in healthy subjects was 186.78 ± 239.31 ρg/ml. The levels of alpha-synuclein oligomer in the saliva of patients with ASD children were 0.13 ± 0.05 ng/ml (p<0.001), and in the healthy subjects was 0.33 ± 0.26 ng/ml. Both alpha-synuclein monomer and alpha-synuclein oligomer levels were low in the saliva of ASD children.

Conclusion

Children with ASD had low levels of alpha-synuclein monomer and oligomer than healthy children which are unique than that of levels found in other degenerative brain diseases.

## Introduction

Autism spectrum disorder (ASD) is a group of disorders that involve brain development in a way that the patients, in their early childhood development, develop two significant behavioral symptoms including social skill problems and restricted, stereotyped behavior. These symptoms appear before the age of three years, and most of these continue for the entire life [1]. The spectrum of ASD represents an entire range of impairments and problems from apparently normal to a mute, disabled child [2]. In the Diagnostic and Statistical Manual of Mental Disorders (DSM-V), the term ASD combines four previous diagnoses, i.e., autistic disorder, Asperger syndrome, childhood dis-integrative disorder, pervasive developmental disorder not otherwise specified (PD-NOS) [3]. In DSM-V, ASD is described as deficits in social communication and restrictive/repetitive behaviors only and no longer requires an impaired language entity for diagnosis [3].

ASD has a prevalence of between 1% and 1.5% globally [4]. ASD is affecting four times more males than females. Although confirmed data is unavailable, the prevalence of ASD is not different in Pakistan than the rest of the world [5]. ASD is a multi-factorial disorder that has both genetic and non-genetic causes [6]. While the exact pathogenesis of ASD is unknown, autoimmune and inflammatory components seem to be involved [7]. However, there is evidence of both neurodegeneration and neuroinflammation in ASD [8]. The histology of the frontal lobe, amygdala, and cerebellum was affected by an unknown inflammatory process [9]. Likewise, the interaction between multiple genes and exposure to environmental factors are responsible for the pathophysiology of ASD [10].

The human synuclein protein family comprises a small (~14kDa), natively unfolded alpha, beta, and gamma synuclein sub-classes. Alpha and beta types are abundant in the central nervous system and presynaptic nerve terminals, whereas gamma type expressed mainly in the peripheral nervous system [11]. Alpha-syn emerges as a significant part of Lewy bodies, which are pathological proteinaceous filamentous structures seen in many neurodegenerative diseases, especially Parkinson’s diseases (PD). In PD, a-synuclein (a-syn) is detectable in the cerebrospinal fluid (CSF), blood, and saliva [12]. Changes in plasma a-syn levels are also observed in children with ASD, which indicate synaptic dysfunctions in these cases [13]. In physiological conditions, a-syn is present in the cells as a monomeric form (a-syn-mono) [14]. In degenerative diseases like PD a-syn-mono becomes abnormally aggregated into a-synuclein oligomers (a-syn-oligo), which is then converted into amyloid fibrils an ultimate precursor of Lewy bodies [15]. This fact establishes that a-syn-oligo is the main neurotoxic form of a-syn [13]. A-syn-oligo is present in CSF, plasma, peripheral nerve fibers, gut, skin, and salivary glands and saliva of PD patients [16]. Saliva is a biological secretion that is easily collectible. Previous studies have investigated synuclein in the saliva of PD patients [17]. The a-syn levels were lower in the saliva of PD patients than in healthy subjects [15]. Recent studies found that salivary levels of a-syn-oligo were higher, whereas a-syn-mono levels were low in PD patients [18].

The diagnosis of ASD is clinical and primarily based on the history, examination, and observations of behavior with the help of scale based questionnaires like childhood autism rating scale-second edition (CARS-2) [19], and diagnostic and statistical manual of mental disorders questionnaire [20]. This helps in early diagnosis and intervention [21]. Early identification of autism helps to offer behavioral therapies to the affected individuals and also reportedly responsible for decreasing family stress and earlier recognition of collateral problems that may present along with significant symptoms of autism [22]. A valid biomarker would help confirm the early clinical diagnosis of ASD. Since a-syn-mono and a-syn-oligo in biological fluids are potential biomarkers, the detection of a-syn in saliva may offer an excellent means of diagnosing the disease. The present study aims to investigate a-syn-mono and a-syn-oligo levels in the saliva of ASD and healthy subjects, and to assess whether these biomarkers can be used to confirm the disease in the future.

## Materials and methods

This is a multi-center study conducted in the department of physiology and multidisciplinary research laboratory of Islamic International Medical College (IIMC), Rawalpindi, in association with the Autism Resource Center (ARC) and Step-to-learn Rehabilitation Center for the slow learners and special children, Rawalpindi. It was a cross-sectional study, and the period of the study was 12 months from August 2018 to August 2019. The ethical review committee of IIMC approved the study. Eighty children (40 ASD affected and 40 healthy controls) were included in this study. Diagnosed cases of ASD and healthy children between three to eight years of age were included in the study. Children having known genetic or chronic illness were excluded from the study.

Sampling technique

A non-probability convenient sampling technique used to collect the required samples. After explaining the procedure and purpose, and obtaining written consent from the parents, the samples were collected. The salivary sample collection was according to the Devic et al. [17] protocol in their study with minor modifications which are as follow:

1. A sample of 2-3 ml of saliva from each ASD patient and healthy subject with 60 minutes fasting was collected by using a small sterilized folded cotton pad and encouraging ASD children to chew it by imitating chewing movements in front of them with the cooperation of their parents/the trainers. Giving enough time in the mouth to be wet. This cotton pad full of saliva was spit into a 50 ml sample collecting vial and then squeezed with gloved hands to get the salivary sample, which was immediately placed in an ice-box to decrease proteolytic activity.

2. Samples centrifuged for 15 minutes at the 2,600 x g at 4°C to get the first clarification, and then again for another 15 minutes at 15,000 x g at 4°C to remove bacteria and contaminate from mucosal cells.

3. After centrifugation, each sample received a protease activity-inhibiting buffer. The samples were then stored at -80°C until the enzyme-linked immunosorbent assay (ELISA) investigation.

4. Monomeric and oligomeric alpha-synuclein levels in the saliva measured using relevant ELISA kits. Salivary a-syn-mono levels are in (pg/ml) and salivary a-syn-oligo levels in (ng/ml).

Data analysis

For statistical analysis, we used the Statistical Package for the Social Sciences (SPSS), version 25 (IBM Corp., Armonk, NY). Results were documented as mean ± SD. The independent samples t-test was applied to analyze the data between the two groups. A p-value of ≤ 0.05 was considered statistically significant.

## Results

A total of 80 children included in the study, (n=40 ASD affected, n=40 healthy controls). The mean age of participating children in both groups was 5.78 ± 1.23 years, and all participants were between four to eight years of age. In ASD groups out of 40 patients, 35 were males and five females. The descriptive analysis showed that ASD is more prevalent in males as compared to females, with a ratio of 7:1 in the ASD group (Table 1).

**Table 1:** Age and gender-related parameters in the study groups *SD=standard deviation

Parameters	Control group, mean ± SD*		Autistic group, mean ± SD*	
Age (years)	5.13 ± 0.85		6.43 ± 0.19	
Gender	Male	Female	Male	Female
	35	05	35	05

**Figure 1:**
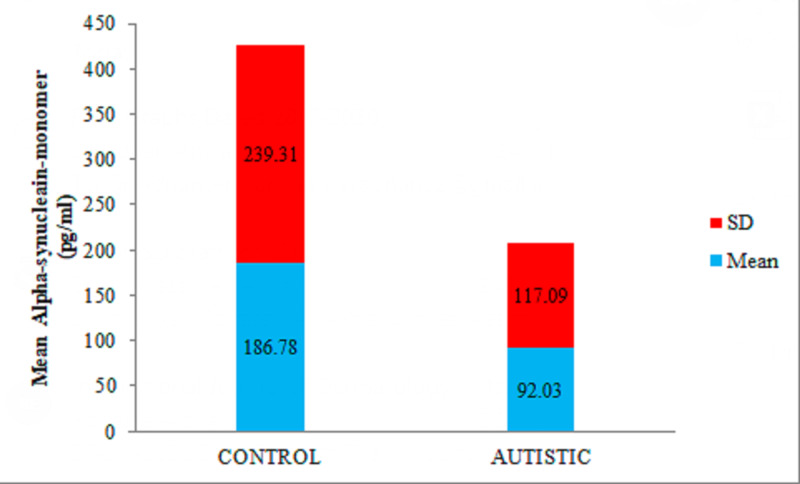
Comparison of the mean ± SD of alpha-synuclein monomer levels in the saliva of control and autistic groups

Statistical analysis to compare the means calculated by the independent-samples t-test revealed significantly lower alpha-synuclein monomer levels in the saliva of patients with ASD than that of healthy subjects. The mean alpha-synuclein monomer levels in the saliva of children with ASD (92.03 ± 117.09 pg/ml) and healthy subjects (186.78 ± 239.31 ρg/ml) having p-value ≤0.05 (Figure 1).

**Figure 2:**
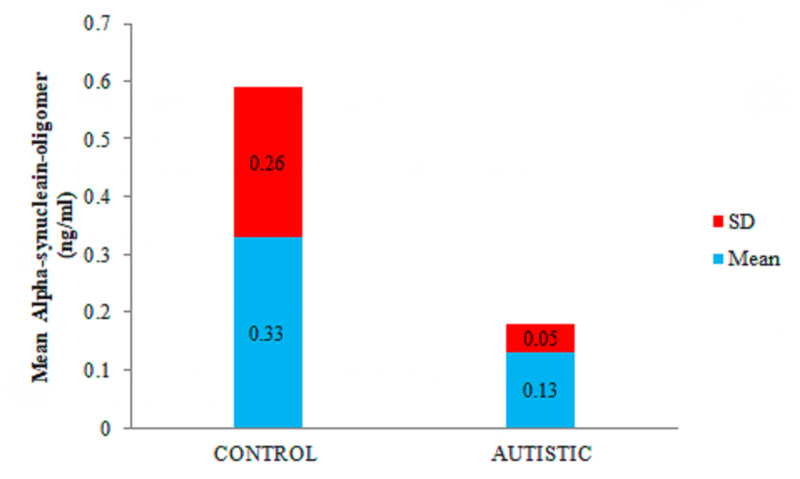
Comparison of the mean ± SD of alpha-synuclein oligomer levels in the saliva of control and autistic groups

The levels of alpha-synuclein oligomer were also low (0.13 ± 0.05 ng/ml) in the saliva of patients with ASD than the salivary levels of the alpha-synuclein oligomer (0.33 ± 0.26 ng/ml) in the healthy subjects having p-value <0.001 (Figure 2). Statistical analysis results have shown p-values of ≤ 0.05 in both groups.

## Discussion

The current study investigates two novel proteins, alpha-synuclein monomer (a-syn-mono), and a-synuclein oligomers (a-syn-oligo), in the saliva of ASD children and uses them as a tool for a better understanding of pathophysiology and early diagnosis. Levels of a-syn-mono and a-syn-oligo assessed by analyzing their presence in the saliva of ASD children of age group four to eight years and comparing them with the healthy controls. The first finding was the reduction in a-syn-mono in the saliva of patients with ASD compared with healthy subjects. This finding is entirely in agreement with two previous observations done on the plasma of ASD children. A study by Kadak et al. [23] found a low serum level of a-syn in ASD children (mean values 33.01 ± 20.78 ng/ml) compared to controls (mean values 241.23 ± 290.5 ng/ml). Sriwimol and Limprasert [13] in another study done on 39 ASD male children, confirms the observation of significantly lower plasma a-syn-mono levels (mean values 10.82 ± 6.46 ng/ml) in the plasma of ASD children compared to healthy controls (mean values 29.47 ± 18.82 ng/ml). The present study on the saliva of 40 ASD children comprising both male and female subjects not only supports the results mentioned above but also establishes the importance and usability of “saliva” as an essential body secretion. Although first used in Parkinson's disease (PD) patients by Devic et al., [17], to the best of our knowledge, this is the first study on ASD children in which saliva is tested for alpha-synuclein-monomer and oligomer levels. The mean value of salivary a-syn-mono in the ASD group found to be 92.03 ± 117.09 pg/ml, whereas, in the control group, it was 186.78 ± 239.31 pg/ml with significant difference (p<0.05). These findings establish that a-syn-mono levels are significantly lower in the saliva of ASD children than the healthy controls.

Although in ASD, the exact cause of the decrease in a-syn-mono level remains unclear as in PD a-syn polymerization, is demonstrated. This explanation suggests that a-syn may spread from cell bodies of salivary neurons via axons to the synaptic terminals around the epithelial cells of salivary glands to saliva. The reduced a-syn-mono concentration detected in the saliva of patients with PD/ASD may be because of an intracellular and axonal polymerization of a-syn in the neurons of the salivary nuclei or ganglia [24]. Many studies showed that ASD patients have abnormal brain growth [25], which may affect the blood-brain and blood-CSF barriers. Therefore, abnormalities of α-synucleins clearance to the CSF and blood may be another cause of decreased levels of α-synuclein in children with ASD.

The second finding is significantly decreased mean salivary level of a-syn-oligo in ASD patients than in healthy subjects. Although a-syn has never been tested in the saliva of ASD patients, this finding is surprising and contrary to the findings previously found in the saliva of PD patients [26] in which there were high levels of a-syn-oligo in the saliva than the healthy controls. In ASD, both oligomer and monomer levels decrease, and this may point towards a defective a-syn-oligo transportation mechanism. Resultant high levels of toxic oligomers can cause local neural damage, and inflammation in areas of the brain, for example, the amygdala, hippocampus, and precuneus, and can be an underlying mechanism of ASD cognitive defects [27]. Another possibility of this low level might be a decrease in the production of a-syn-mono, resulting in decrease aggregation.

In vitro-generated a-syn-oligo induce transmembrane seeding of a-syn aggregation in neurons [28]. Thus a-syn-oligo can induce intracellular a-syn aggregation and could thus spread the disease between inter-connected cells. Low levels of both monomeric and oligomeric form in extracellular fluids can be because of their increased seeding in the tissues somewhere else in the body. Another explanation could be an a-syn-oligo molecule in many forms [29] that may lead to the wide variability of a-syn-oligo conformation in the saliva of ASD and its extracellular levels.

Currently, the diagnosis of ASD is based only on clinical assessment, and no biochemical test is available to speed up the diagnostic process. Early diagnosis and prompt behavioral therapy are the cornerstones to the successful management of ASD. The clinical variabilities in ASD children have encouraged increasing interest in biomarker detection as potentially useful indicators for diagnosis and treatment. Many studies identified potential blood-based biomarkers for ASD, including brain-derived neurotrophic factor (BDNF) [18], neurotrophin-4 (NT-4) [30], and the glial fibrillary acidic protein. However, no biomarkers to date have been able to show high sensitivity and specificity. The present study found that salivary alpha-synucleins levels variations are equivalent to the plasma changes seen in ASD children; with the use of saliva to determine the a-syn levels is an effort to ease down the invasive blood and CSF sampling options.

There were few limitations like small sample size and synuclein levels not tested in the blood simultaneously to the study performed under the existing resources and facilities. Further elucidation about the alpha-synuclein level needs to be investigated, on a larger scale and in a different study setting, to confirm these findings and compare them against the severity of the ASD symptomatically.

## Conclusions

This study concluded that the children with ASD have low levels of both a-syn-mono and a-syn-oligo than healthy children and establishes the importance of saliva as an easily collectible and non-invasive diagnostic tool than blood and CSF in detecting alpha-synuclein in ASD. Measured together, salivary a-syn-mono and oligomer levels are appropriate biochemical markers for early diagnosis of ASD.
